# Supplementary material for: Estimated Healthcare Costs of Melanoma and Keratinocyte Skin Cancers in Australia and Aotearoa New Zealand in 2021
Source: Int J Environ Res Public Health. 2022 Mar 8;19(6):3178. doi: 10.3390/ijerph19063178 (PMC8948716; doi:10.3390/ijerph19063178)
Supplement: Supplementary file 1 [file ijerph-19-03178-s001.zip › ijerph-1570091-SI.pdf]

Supplementary File

Table S1: Model inputs for melanoma diagnosis and treatment pathways

	Australian inputs AU$			NZ inputs NZ$		
**Description**	**Value**	**Low**	**High**	**Value**	**Low**	**High**
**Costs**						
GP consult	37.05	37.05	38.20	119.84	All costs varied ±20%	
Biopsy	37.35	17.40	44.40	71.43		
Pathology	82.30	73.40	82.60	140.00		
Surgical excision	239.65	211.45	271.95	458.33		
Skin graft	304.55	241.70	355.35	582.45		
Excision - benign lesion (thought to be melanoma)	93.25	59.90	115.65	178.34		
Specialist consult	72.75	36.55	138.40	250.00		
BRAF mutation test	196.35	173.25	196.35	375.52		
Complete lymph node dissection	16,303	14,673	17,933	31,179		
FDGPET scan	999	899	1099	1,911		
Hospitalisation for melanoma (procedure / AEs)	3,154	2,523	3,785	7,701		
Palliative care	11,835	10,652	13,019	22,634		
Radiotherapy course of treatment	7,962	7,166	8,758	15,227		
SLNB	5,818	5236	6400	11,127		
Dabrafenib and trametinib	336,829	269,463	404,195	n/a		
Ipilimumab	144,382	94,950	153,309	n/a		
Nivolumab	48,851	14,449	55,057	68,379		
Pembrolizumab	102,430	27,288	135,228	81,120		
Interferon therapy	n/a	n/a	n/a	16,920		
**Number needed to biopsy**						
Number needed to biopsy for GPs	14.6	8.5	17	NZ values are the same for Australia<br><br>Adjuvant stage III therapies & Ipilimumab therapy excluded		
Number needed to biopsy for specialists	3.5	2.5	7.5			
**Probabilities**						
Invasive melanoma is stage IA	0.121	All probabilities varied ±10% except probability of stage III resectable and stage III/IV unresectable varied ±20%				
Invasive melanoma is stage IB or II	0.242					
Invasive melanoma is resectable stage III	0.101					
Invasive melanoma is unresect. stage III/stage IV	0.036					
Suspected melanoma was excised and pathology showed benign	0.194					
Skin graft with excision	0.512					
Re-excision required	0.05					
CLNB	0.20					
SLNB in those eligible	0.604					
Positive nodes after SLNB	0.18					
Adjuvant radiotherapy in stage III	0.05					
Stage IB and II seen by a specialist	0.495					
Multiple melanomas	0.008					
Subsequent melanoma that is stage IA	0.816					
Patient has stage III resect and dabraf/tram	0.084					
Patient has ipilimumab	0.217					
Patient has nivolumab	0.529					
Patient has pembrolizumab	0.197					
Patient has dabrafenib and tremetinib	0.057					
Serious AEs dab/tram, ipi, nivo, pembro	0.15-0.28					
4-year survival with stage III adjuvant nivolumab	0.779					
5-year survival with stage III adjuvant dab/tram	0.52					
5-year survival from dabrafenib and trametinib	0.34					
5-year survival from ipilimumab[1]	0.26					
5-year survival from nivolumab	0.39					
5-year survival from pembrolizumab	0.41					
7-year survival from stage III (without adjuv therapy)	0.53					

Australian hospitalisation cost estimate was the average of items for skin malignancy DRGs in the CosQ and Cancer Cost Model datasets (Supplementary File) (higher cost than KCs for DRG with complications).

The New Zealand estimates for GP consult (annual capitation rate), hospital costs (Casemix DRG data), nivolumab & pembrolizumab, (PHARMAC), pathology (PathLab) and specialist consult (PHARMAC) were locally sourced while the remainder applied a multiplier of 1.91 to the Australian values (mean inflator of 8 KC and melanoma items compared with Australian prices). A blanket 20% higher and lower value to costs were applied to the NZ cost estimates in one-way sensitivity analyses and 10% for all probabilities.

Rate to Prob formulas were applied to get an annual probability for survival rates for advanced melanoma therapies. For ipilimumab, 0.58 was applied in 1st year.

Table S2: Model inputs for KC diagnosis and treatment pathways

	Australian estimates			New Zealand estimates[1]		
**Description**	**Value**	**Low**	**High**	**Value**	**Low**	**High**
**Costs[2]**	**AU$**			**NZ$**		
GP consultation for skin check or treatment	37.05	36.30	38.20	140.88	All costs varied ±20%	
Skin biopsy	43.10	21.05	44.40	85.49		
Shave biopsy	105.40	53.70	107.40	209.06		
Excision of KC	136.75	104.55	181.05	271.24		
Skin graft with KC excision	319.90	241.70	355.35	634.52		
Histopathology of skin tissue	73.95	73.40	82.60	140.00		
Excision of a benign lesion (thought to be malignant)	81.15	57.85	113.85	160.95		
Cryotherapy	41.10	20.55	41.10	81.62		
Imiquimod	93.58	79.79	124.65	21.72		
Specialist consultation	72.75	36.55	127.45	250.00		
Hospitalisation treatment for KC[3]	2,903.00	2,322.40	3,483.60	5,429.50		
Mohs surgery	498.78	435.65	544.55	989.32		
Radiotherapy treatment of KC	155.50	147.50	212.00	308.43		
**Probabilities[2]**						
Excision after a biopsy confirming KC	0.905	0.724	1.086	All NZ probabilities are the same as those for Australia		
Curettage or shave biopsy with curative intent	0.029	0.026	0.032			
Surgical excision after shave biopsy	0.731	0.658	0.804			
Punch biopsy	0.380	0.322	0.422			
Punch biopsy pathology returns benign lesion	0.162	0.146	0.178			
Excision pathology returns benign lesion	0.050	0.045	0.055			
Skin graft/flap connected with an excision	0.176	0.141	0.211			
At least one KC treated within 12 months	0.192	0.173	0.211			
Mean number of KCs in those >1 within 12 months	4	4.4	3.6			
Re-excision for unclear margins or recurrence[4]	0.03	0.02	0.04			
A person is treated for benign and KC lesions[6]	65.2% over 10 years					
Treating a benign lesion with cryotherapy	0.657	0.591	0.723			
Cryotherapy if non-surgically treated	0.719	0.575	0.863			
GP refers patient to hospital for treatment[5]	0.030	0.025	0.035			
Mohs surgery if a specialist is seen	0.069	0.055	0.083			
Skin graft with mohs surgery	0.289	0.231	0.347			
Person has multiple skin cancers after first year[6]	50.9% over 5 years					
Radiotherapy after excision	0.029	0.023	0.035			
Person sees a specialist	0.065	0.059	0.072			
Excision after seeing a specialist	0.889	0.845	0.933			
More frequent follow up - 6 monthly	0.250	0.2	0.3			

1. The New Zealand estimates for GP consult (annual capitation rate), hospital costs (Casemix DRG data), imiquimod (Pharmac), pathology (PathLab) and specialist consult (Pharmac) were locally sourced while the remainder applied a multiplier of 1.91 to the Australian values (mean inflator of 8 KC and melanoma items compared with Australian prices). A blanket 20% higher and lower value to costs were applied to the NZ cost estimates in one-way sensitivity analyses.
2. Unless otherwise specified, estimates were derived from the QSkin patient-level dataset 2010-2020. The low and high values of Australian costs were the 25th and 75th percentiles. The high and low values for probabilities were varied 5-20%, lower if the value was closer to 100%.
3. Australian hospitalisation cost estimate was the average of items for skin malignancy DRGs in the CosQ and Cancer Cost Model datasets (Supplementary file).
4. Clinical expert estimate was used for the probability of re-excision rate, both occurring infrequently.
5. Source: AIHW Skin Cancer in Australia, rate to probability of 44 per 10,000 hospitalisation rate for KC (age-standardised, ICD-C44).

6. Rate to probability formulas were applied to get an annual probability from a 10 year rate. A person treated with both benign and KC lesions was 0.65175 over 10 years. A person treated with multiple KCs was 0.54705 over 10 years.

Table S3. Medicare MBS and PBS codes[1] included in the skin cancer cost calculations

	Biopsy / consults	Excision codes	Non-excision codes	Skin flap/graft codes	Pathology codes	PBS items codes
KC	30071<br><br>23<br>36<br>104<br>105<br>107<br>108<br>110	31255, 31256; 31257; 31258; 31260; 31261; 31262; 31263; 31265; 31266; 31267; 31268; 31270; 31271; 31272; 31273; 31275; 31276; 31277; 31278; 31280; 31281; 31282; 31283; 31285; 31286; 31287; 31288; 31290; 31291; 31292; 31293; 31295; 31000; 31001; 31002; 31356; 31358; 31359; 31361; 31363; 31365; 31367; 31369; 31340	30196; 30197; 30202; 30203; 30205;	45200; 45203; 45206; 45207 45000; 45003; 45400; 45403; 45239; 45442; 45445; 45448; 45451; 45665;	72813; 72816; 72817; 72823; 72824; 72825; 72826; 72830; 72836; 72818; 72827; 72828; 72838	Imiquimod: 4559Y; 2637T; 2546B;<br>5-FU fluorouracil: 4222F<br>Sonidegib: 11304Y[2]<br>Vismodegib: 11070P[2]
Melanoma		31300; 31305; 31310; 31315; 31320; 31325; 31330; 31335; 31371; 31372; 31373; 31374; 31375; 31376	PET: 61553<br>BRAF test: 73336			See below
Lesion		31230; 31235; 31240; 31205; 31220; 31225; 31210; 31215; 31357; 31360; 31362; 31364; 31366; 31368; 31370	30192; 30195;			Imiquimod = 4134N; 10106X;

1. MBS and PBS codes have changed over time, here all the codes are listed but some are no longer on the Schedules.
2. These codes are for metastatic or locally-advanced BCC which is very rare and not included in the KC pathways model in this study.

Table S4: PBS codes for melanoma pharmacotherapies[1]

Listed	Drug	ATC5	Unresectable stage III & IV PBS item	Resectable stage III & IV PBS item
Aug-13	Ipilimumab	L01XC11	2638W	
			2641B	
Dec-13	Dabrafenib[2]	L01XE23	2963Y	11823G
			2846T	11820D
			2954L	
			10003L	
Sep-15	Pembrolizumab	L01XC18	10475H	12125E
			10493G	12127G
			10424P	12130K
			10436G	12120X
			12122B	
			12123C	
			12124D	
			12128H	
Aug-15	Trametinib	L01XE25	10403M	11821E
			10382K	11819C
			10385N	
			10405P	
May-16	Nivolumab	L01XC	10775D	11906P
			10764M	11900H
			10745M	
			10748Q	
			11532Y	
			11543M	
Apr-17	Cobimetinib[3]	L01XE38	11074W	
			11075X	
Apr-17	Vemurafenib	L01XE15	11076Y	
			11081F	

1. Blue shaded cells denote initial treatment, the remainder are continuing prescriptions
2. dabrafenib + trametinib must be prescribed together
3. vemurafenib + cobimetinib must be prescribed together

Table S5: Distribution types and parameters used in the model

Melanoma			
	Type	Para 1	Para 2
**Australian Costs**			
CLND		44.44	0.0027
Ipilumumab		44.44	0.0003
NNB for GPs		44.44	3.0441
SLNB		44.44	0.0076
Dabrafenib/trametinib	Gamma	44.44	0.0001
NNB for specialists	Para 1=α<br>Para 2=λ	44.44	12.6984
Hospitalisation visit		44.44	0.0141
Palliative care		44.44	0.0038
Nivolumab		44.44	0.0009
Pembrolizumab		44.44	0.0004
Radiation		44.44	0.0056
**Probabilities**			
Stage III disease		39.85	354.7450
Stage III/ IV disease		42.81	1146.3150
Stage IB & II disease		33.45	104.7634
SLNB		67.06	44.0491
Early stage for subs melanoma		5.23	0.8296
Radiation therapy		42.17	801.2722
Re-excision	Beta	42.17	801.2722
Stage IA disease	Para 1=α<br>Para 2=β	38.95	282.9193
Ipilimumab		34.58	124.7857
Nivolumab		20.40	18.1672
Pembrolizumab		35.49	144.6700
Dabrafenib/trametinib		41.85	692.4285
Dabrafenib/trametinib adjuvant therapy		40.63	443.0290
Stage IIIA disease		34.46	122.4789
CLND		35.36	141.4222
**New Zealand costs**			
CLND		44.4444	0.0014
NNB for GPs		44.4444	3.0441
SLNB		44.4444	0.0040
Dabrafenib/trametinib		44.4444	0.0002
NNB for Specialists	Gamma	44.4444	12.6984
Palliative care		44.4444	0.0020
Nivolumab	Para 1=α<br>Para 2=λ	44.4444	0.0006
Pembrolizumab		44.4444	0.0005
Hospitalisation visit		44.4444	0.0058
Radiation		44.4444	0.0029
Interferon therapy		44.444	0.2627

Keratinocyte cancer			
	Type	Para 1	Para 2
**Probabilities**			
Biopsy and excision		194.10	20.45
Multiple KCs <12 mths		35.71	150.13
Benign & cryotherapy		69.28	36.12
Benign		37.05	190.79
Punch biopsy		126.15	205.82
Cryotherapy		56.55	22.06
Skin graft/flap		36.47	171.33
Freq of follow up		33.08	99.25
Hospitalisation	Beta	44.24	10033.56
Mohs surgery	Para 1=α<br>Para 2=β	41.33	561.70
Skin graft/flap with Mohs		31.33	77.23
Multiple KCs over time		16514	8410
On pathology - benign		42.17	801.27
Radiotherapy		43.14	1465.41
Re excision		43.08	1392.96
Shave biospy		145.28	361.46
Specialist visit		41.49	596.83
Specialist excision		4.05	0.51
Shave biopsy and surgery		11.33	4.22
**Counts**			
No. KCs if multiple (log normal distribution)	Para 1=μ<br>Para 2=σ	1.28	0.46

Acronyms: CLND = complete lymph node dissection; GP = general practitioner; KCs = keratinocyte cancers; NNB = number needed to biopsy; para = parameter; SLNB = sentinel lymph node biopsy,

Explanatory notes on analysis of QSKIN data and other published data sources

Using the codes applicable to skin cancer services on the MBS and PBS, an analysis of the total counts of each service was aggregated for excisions, shave biopsy and curettage, cryotherapy, Mohs surgery and radiotherapy with KC excision or shave biopsy. Service data (long) was converted to person-level data (wide) in STATA V15.

Among all persons with codes identifying the person was treated for KC (16,514), 14940 or 90.47% had at least 1 surgical excision. 17.1% of persons had their 1st and 2nd lesion excised on the same day and this proportion was similar for those with 2nd and 3rd lesions excised, 3rd and 4th lesions excised etc. 19.2% had multiple KCs within 12 months. Those with at least 1 dermatologist visit were 19.5%, and 13.7% saw a plastic surgeon. Of those who saw a specialist, 6.5% had Mohs surgery. Only 1 person was treated with metastatic BCC and was treated with sonidegib. 4.01% were treated with imiquimod and 25.3% had had skin graft or flap.

Summary of treatments and services for KCs from 2011 to 2020

KCs	Total services	Total persons	% all persons
Excisions	60004	14940	90.47%
Shave biopsy & curettage	19738	4735	28.67%
Cryotherapy	4252	1700	10.29%
Mohs surgery	482	298	1.80%
Skin graft or flap	7546	4172	25.26%
Biopsy	71263	12976	78.58%
GP visits	1145256	16503	99.93%
Dermatologist	33299	3212	19.45%
Plastic surgeon	13459	2259	13.68%
Imiquimod	1125	663	4.01%
Sonidegib	12	1	0.01%
Radiotherapy and KC exc or shave		473	2.86%

Conditional probabilities were assessed to obtain necessary annual probabilities for the model; a subset of people with 1 excision (1370) were assessed for those who had a biopsy 520/1370 (38.0%) and those who saw either a dermatologist or plastic surgeon and had an excision 4050/4556 (88.9%) and proceeded to Mohs surgery 298/4348 (6.9%). A total of 2622/14940 (17.6%) had a skin graft with an excision and 86/298 (28.9%) had one with Mohs surgery. A total of 2585/3548 (72.9%) had an excision after a shave biopsy.

Using service-level data, descriptive statistics (mean, sd, 25th pctile, 75th pctile, median, minimum and maximum) were generated for costs of individual services for excision, cryotherapy, grafts, biopsy, GP consults, dermatologist and plastic surgeon consults, imiquimod, pathology, shave biopsy, benign excision and radiotherapy. The model applied the mean cost and the 25th and 75th percentiles in one-way sensitivity analyses. We assumed that 3% of KCs excised would undergo re-excisions for unclear margins, partially confirmed by expert opinion and assessment of residual or recurrent KCs MBS codes used between 2011-2020 but are now obsolete (MBS items: 31256-31295). Re-excisions are now billed using the existing excision codes.

We derived the costs for KCs and melanoma episodes in hospital from two datasets [1, 2] providing hospital admissions and associated costs using ICD codes C430-439 and C440-449. Costs were per hospital separation of admissions for KCs and melanoma. The probability of hospitalisation for KC treatment is small and reported by AIHW as 44 per 10,000 persons (age standardised) nationally [3]. The hospitalisation rate has been stable over time for men (58 per 10,000) and women (32 per 10,000) [3]. A rate to probability formula was applied and the annual probability applied was 0.00439.

Melanoma costs also used service-level data and descriptive statistics for melanoma-related codes in the MBS data. This included the cost for excising a melanoma, a benign lesion (believed to be a melanoma), biopsy, pathology, GP and specialist visits, BRAF test, skin graft and flap. The model applied the mean cost and the 25th and 75th percentiles for one-way sensitivity analyses. The cost for complete lymph node dissection, sentinel lymph node dissection, a course of radiotherapy, palliative care were derived from the latest National Hospital Cost Data Collection Round 22 report [4].

We analysed person-level costs in QSKIN for persons with advanced melanoma (n=109), identified by the pharmacotherapies received and only available to treat stage III and IV melanomas. The model applied the mean cost and the 25th and 75th percentiles for one-way sensitivity analyses. The costs for ipilimumab over 1 year were available for 21 patients, 36 patients for nivolumab, 43 patients with pembrolizumab, 5 people for dabrafenib and trametinib. The proportions receiving each therapy were derived from publicly available PBS Item Reports for 2020-21 full financial year. In unresectable disease, nivolumab was used for 52.9%, pembrolizumab for 19.7%, ipilimumab for 21.7% and 5.7%for dabrafenib/trametinib, based on the number of scripts for initial treatments (excludes continuing scripts). 90% of patients received treatments within 12 months and costs were applied within the first year in the model with 10% carrying over into the second year. It was assumed that a person with unresectable stage III or IV melanoma would have a multidisciplinary team involved in their management with costs assigned for a 4-specialist consultation.

Rates of SLNB uptake are highly variable ranging from 33% to 64% in studies from 2006 to 2018 [5]. A rate of 60.4% was applied from Neibling *et al.* (2014) in a series of 5011 patients in Sydney [6]. The proportion of patients with SLNB that were positive were 18% [7].

Staging is particularly uncertain in Australia as this is not available from cancer registry data and is not mandated when reporting new cases of cancer to the registry. The AJCC staging system has also changed and this may affect proportions in each category. National estimates of staging from Cancer Australia omit melanoma *in situ*. In a large series of melanoma in Queensland (n=97114), melanoma *in situ* was estimated in 2017 as 44.8% of all cases of melanoma [8] but there is evidence this is rising steeply over time due to greater awareness and skin checking [9]. In the model, we used a large series of patients from Europe, US and Australia used to document the AJCC 8th Staging system [10]. This included the stages IA to IIID. In situ cases were assumed to be 50% of invasive cases and stage IV was from NSW Cancer Institute records

Stage	N	%	Source
In situ	21623	50.0%	Assumed
IA	5225	12.1%	Gershenwald 2017 [10]
IB&II	10466	24.2%	
IIIA-C	4377	10.1%	
IIID	205	0.5%	
IV	1350	3.1%	Estimated
total	43246	21623	
Final proportions applied in model:			

Stage	N	%	Source
In situ	0.500		
IA	0.121		
IB&II	0.242		
IIIA-C	0.101		
IIID/IV	0.036		

Australian Bureau of Statistics Life Tables 2017-2019 for Australia (Table 1.9) were used for generating probabilities of death by age in the general population, to represent background mortality in the model.

Three sources were used for assessing the number needed to biopsy (NNB). Within a larger systematic review, Nelson *et al.* 2019 reported the Australian NNB among GPs as 14.6, an Australian national audit study with a large number of GPs (n=193) [11]. For dermatologists, 3.5 was applied from Chia *et al*. 2008, an Australian study with 35 dermatologists [12].

Figure S1: Results of 1-way sensitivity analysis (Australia)

a) Melanoma

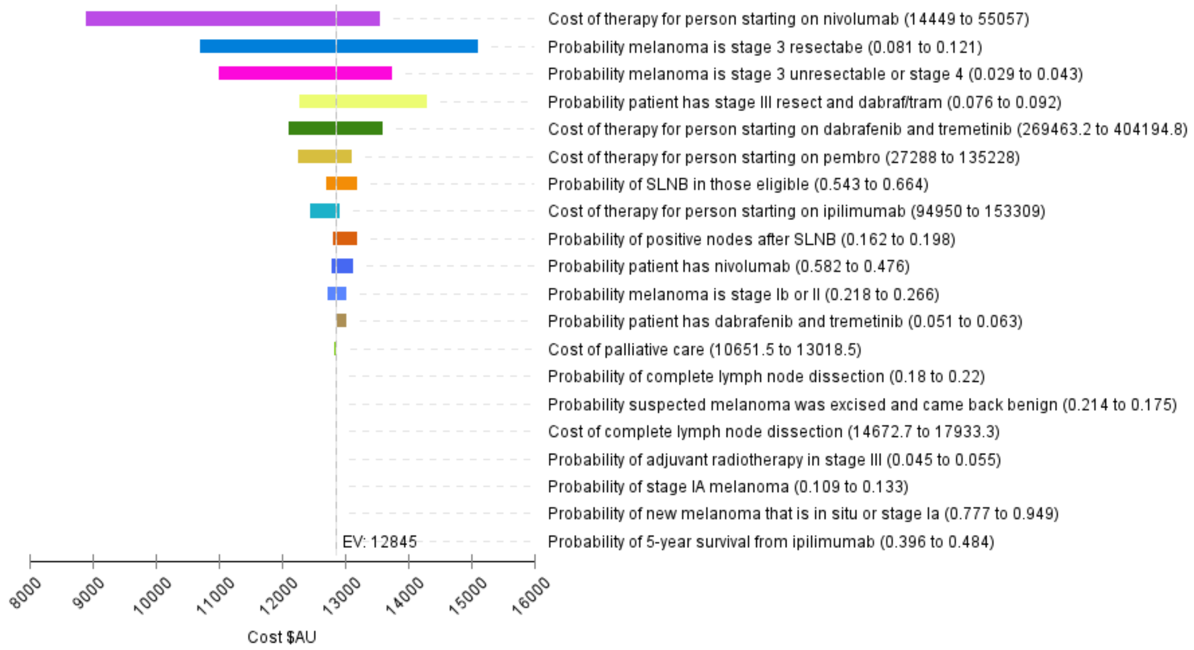

b) KC

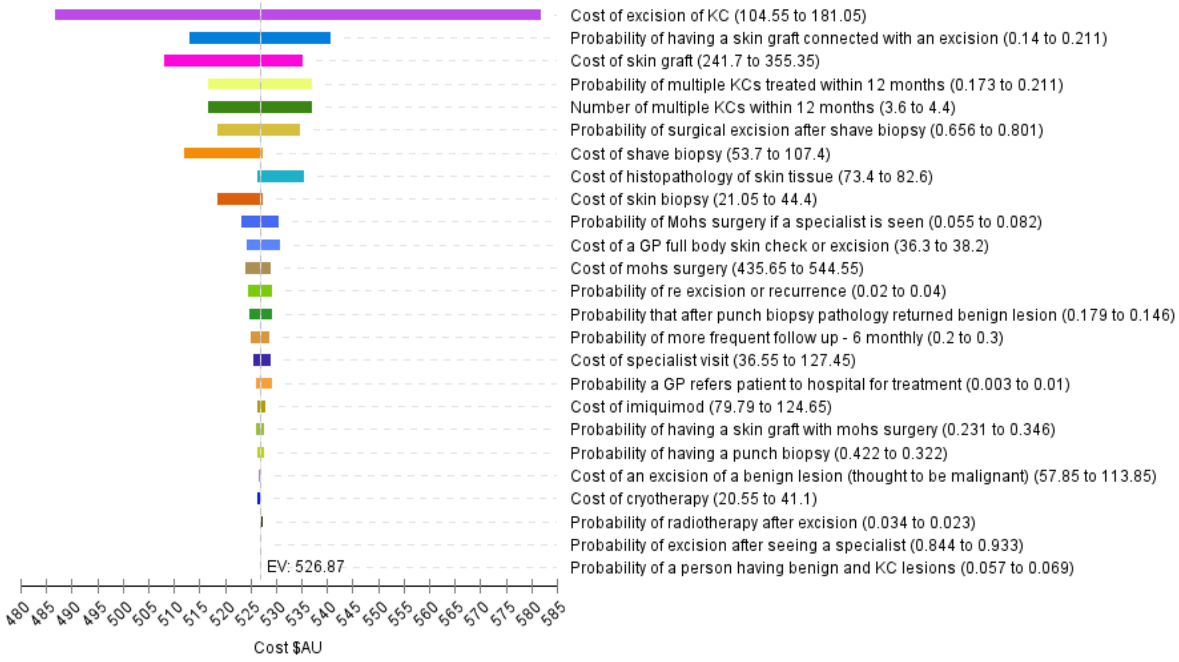

Figure S2: Results of 1-way sensitivity analysis (NZ)

a) Melanoma

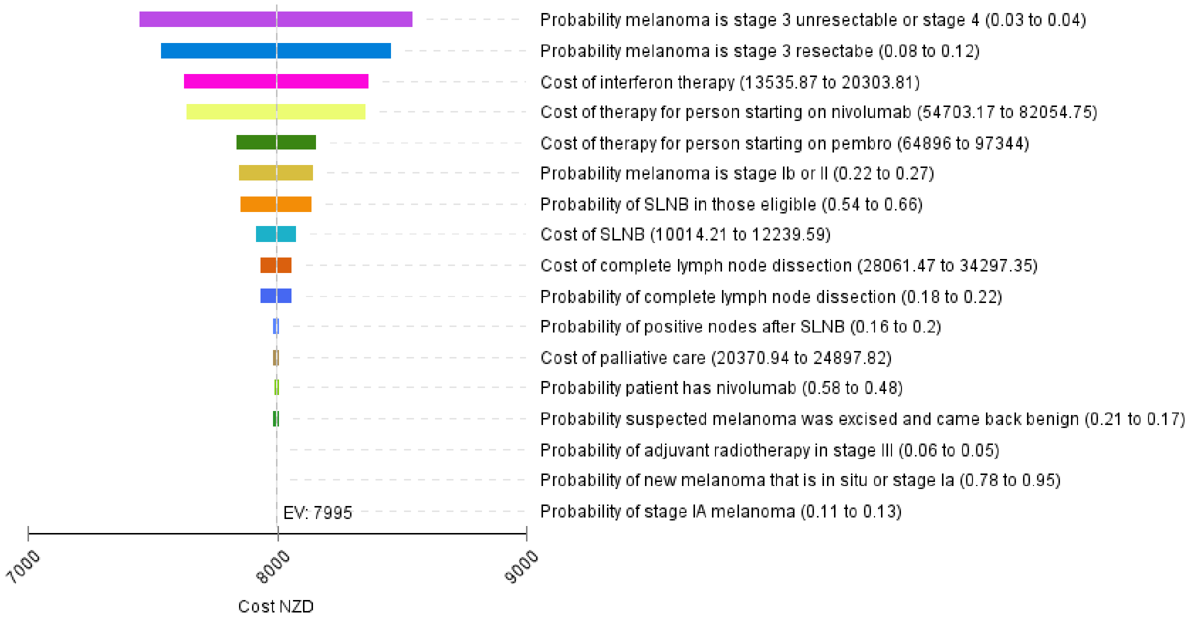

b) KC

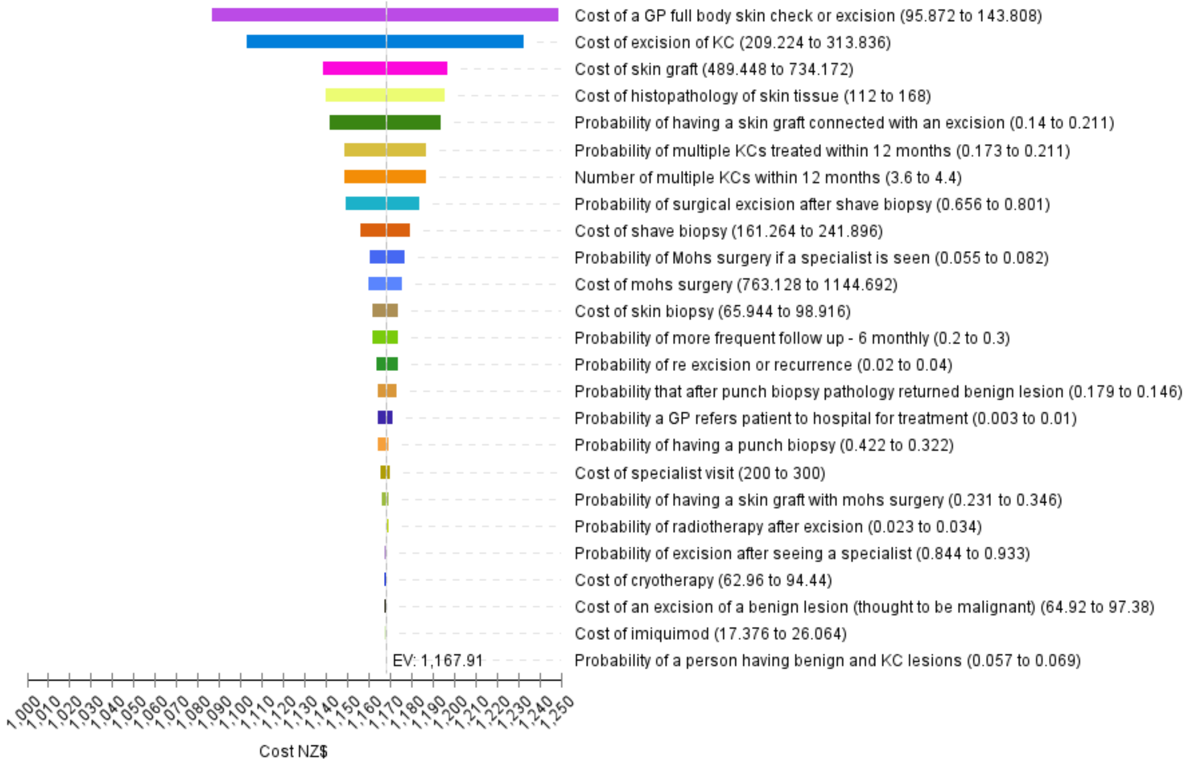

References

1. Bates, N.; Callander, E.; Lindsay, D.; Watt, K., CancerCostMod: a model of the healthcare expenditure, patient resource use, and patient co-payment costs for Australian cancer patients. *Health Econ Rev.* **2018,** 8, (1), 28. doi: 10.1186/s13561-018-0212-8.
2. Merollini, K. M. D.; Gordon, L. G.; Aitken, J. F.; Kimlin, M. G., Lifetime Costs of Surviving Cancer-A Queensland Study (COS-Q): Protocol of a Large Healthcare Data Linkage Study. *Int J Environ Res Public Health.* **2020,** 17, (8), 2831. doi: 10.3390/ijerph17082831.
3. Australian Institute of Health and Welfare, Skin cancer in Australia Cat. no. CAN 96. In AIHW: Canberra, 2016.

4. Independent Hospital Pricing Authority, National Hospital Cost Data Collection Report, Public Sector, Round 22 (Financial year 2017-18) In Sydney, 2020.
5. Rapport, F.; Smith, A. L.; Cust, A. E.; Mann, G. J.; Watts, C. G.; Gyorki, D. E.; Henderson, M.; Hong, A. M.; Kelly, J. W.; Long, G. V.; Mar, V. J.; Morton, R. L.; Saw, R. P.; Scolyer, R. A.; Spillane, A. J.; Thompson, J. F.; Braithwaite, J., Identifying challenges to implementation of clinical practice guidelines for sentinel lymph node biopsy in patients with melanoma in Australia: protocol paper for a mixed methods study. *BMJ Open.* **2020,** 10, (2), e032636. doi: 10.1136/bmjopen-2019-032636.
6. Niebling, M. G.; Haydu, L. E.; Karim, R. Z.; Thompson, J. F.; Scolyer, R. A., Pathology review significantly affects diagnosis and treatment of melanoma patients: an analysis of 5011 patients treated at a melanoma treatment center. *Ann Surg Oncol.* **2014,** 21, (7), 2245-51. doi: 10.1245/s10434-014-3682-x. Epub 2014 Apr 19.
7. Leeneman, B.; Schreuder, K.; Uyl-de Groot, C. A.; van Akkooi, A. C. J.; Haanen, J.; Wakkee, M.; Franken, M. G.; Louwman, M. W. J., Stage-specific trends in incidence and survival of cutaneous melanoma in the Netherlands (2003-2018): A nationwide population-based study. *Eur J Cancer.* **2021,** 154:111-119., (doi), 10.1016/j.ejca.2021.06.007. Epub 2021 Jul 10.
8. Aitken, J. F.; Youlden, D. R.; Baade, P. D.; Soyer, H. P.; Green, A. C.; Smithers, B. M., Generational shift in melanoma incidence and mortality in Queensland, Australia, 1995-2014. *Int J Cancer.* **2018,** 142, (8), 1528-1535. doi: 10.1002/ijc.31141. Epub 2017 Nov 21.
9. Coory, M.; Baade, P.; Aitken, J.; Smithers, M.; McLeod, G. R.; Ring, I., Trends for in situ and invasive melanoma in Queensland, Australia, 1982-2002. *Cancer Causes Control.* **2006,** 17, (1), 21-7. doi: 10.1007/s10552-005-3637-4.
10. Gershenwald, J. E.; Scolyer, R. A.; Hess, K. R.; Sondak, V. K.; Long, G. V.; Ross, M. I.; Lazar, A. J.; Faries, M. B.; Kirkwood, J. M.; McArthur, G. A.; Haydu, L. E.; Eggermont, A. M. M.; Flaherty, K. T.; Balch, C. M.; Thompson, J. F., Melanoma staging: Evidence-based changes in the American Joint Committee on Cancer eighth edition cancer staging manual. *CA Cancer J Clin.* **2017,** 67, (6), 472-492. doi: 10.3322/caac.21409. Epub 2017 Oct 13.
11. Nelson, K. C.; Swetter, S. M.; Saboda, K.; Chen, S. C.; Curiel-Lewandrowski, C., Evaluation of the Number-Needed-to-Biopsy Metric for the Diagnosis of Cutaneous Melanoma: A Systematic Review and Meta-analysis. *JAMA Dermatol.* **2019,** 155, (10), 1167-1174. doi: 10.1001/jamadermatol.2019.1514.
12. Chia, A. L.; Simonova, G.; Dutta, B.; Lim, A.; Shumack, S., Melanoma diagnosis: Australian dermatologists' number needed to treat. *Australas J Dermatol.* **2008,** 49, (1), 12-5. doi: 10.1111/j.1440-0960.2007.00410.x.
